# Using Mobile Apps to Promote a Healthy Lifestyle Among Adolescents and Students: A Review of the Theoretical Basis and Lessons Learned

**DOI:** 10.2196/mhealth.3559

**Published:** 2016-05-05

**Authors:** Denise Jantine Dute, Wanda Jose Erika Bemelmans, João Breda

**Affiliations:** ^1^ National Institute for Public Health and Environment (RIVM) Bilthoven Netherlands; ^2^ World Health Organization Regional Office for Europe Noncommunicable Diseases and Life-Course Copenhagen Denmark

**Keywords:** mHealth, mobile phones, behavior change, health promotion, physical activity, nutrition, overweight, adolescents and students

## Abstract

**Background:**

European adolescents and students tend to have low levels of physical activity and eat unhealthy foods, and the prevalence of overweight and obesity has increased, which poses a public health challenge. Mobile apps play an important role in their daily lives, suggesting their potential to be used in health-promoting strategies.

**Objective:**

This review aimed to explore how mobile apps can contribute to the promotion of healthy nutrition, physical activity, and prevention of overweight in adolescents and students. For the apps identified, the review describes the content, the theoretical mechanisms applied, and lessons learned.

**Methods:**

The databases Scopus, MEDLINE, Embase, and PsycINFO were searched for English-language publications from January 2009 to November 2013. Studies were included if (1) the primary component of the intervention involves an app; (2) the intervention targets healthy nutrition, or physical activity, or overweight prevention; and (3) the target group included adolescents or students (aged 12-25 years).

**Results:**

A total of 15 studies were included, which describe 12 unique apps. Ten of these apps functioned as monitoring tools for assessing dietary intake or physical activity levels. The other apps used a Web-based platform to challenge users to exercise and to allow users to list and photograph their problem foods. For 5 apps, the behavioral theory underpinning their development was clearly specified. Frequently applied behavior change techniques are prompting self-monitoring of behavior and providing feedback on performance. Apps can function self-contained, but most of them are used as part of therapy or to strengthen school programs. From the age of 10 years users may be capable of using apps. Only 4 apps were developed specifically for adolescents. All apps were tested on a small scale and for a short period.

**Conclusions:**

Despite large potential and abundant usage by young people, limited research is available on apps and health promotion for adolescents. Apps seem to be a promising health promotion strategy as a monitoring tool. Apps can enable users to set targets, enhance self‐monitoring, and increase awareness. Three apps incorporated social features, making them “social media,” but hardly any evidence appeared available about their potential.

## Introduction

The lifestyle of European adolescents and students poses a serious public health challenge. Adolescents and students tend to have low levels of physical activity and eat unhealthy foods. In addition, the prevalence of overweight and obesity within this group has increased in many countries. A study that monitored the physical activity of children and adolescents at ages 9, 11, 12, and 15 years found that physical activity levels decreased as children enter adolescence [1]. The Health Behavior in School-aged Children 2009/2010 survey showed that young people in Europe prevalently skip breakfast and consume increasing amounts of soft drinks and less amounts of fruits between ages 11 and 15 years [2]. Furthermore, the prevalence of overweight, including obesity, among 11-year-old European children ranged from 5% to more than 26% in some countries [2]. The annual overweight rates are increasing continuously, which demonstrates the need for public intervention [3].

Mobile apps seem to be promising tools to help people improve their health [4]. Apps are software applications that enable programs to run on smartphones. Because smartphones can be used anywhere and at any time, they can potentially reach many people and can offer good opportunities to contribute to health promotion and health protection. They can be particularly beneficial in reaching adolescents and students. In 2012, 56% of Dutch adolescents (12-15 years old) [5,6] and 78% of Dutch students (16-25 years old) owned a smartphone [7]. Young people and middle-aged adults use their smartphones more often than older adults. A total of 58% of Europeans aged 16-24 years use mobile Internet compared with 36% and 12% of the population aged 25-54 years and 55-74 years, respectively [8].

An interesting question is whether apps can be applied in health promotion for adolescents and students. Additionally, it would be beneficial to know how mobile apps can be used effectively in health promotion. So far, little research has been published about their effectiveness in health promotion [9]. Crutzen et al [10] found that Web-based interventions embedded in existing structures, such as health care or schools, are more effective compared with Web-based interventions alone. Furthermore, evidence indicates that effectiveness of health promotion interventions also depends on the extent to which they are based on theoretical mechanisms for changing behavior [11,12].

Theory-based interventions use one or more theories during their development [13]. Several theories have been proposed that can predict and explain human behavior. For example, the theory of planned behavior describes 3 factors, namely, attitude, subjective norm, and perceived behavioral control, that have an influence on an individual's intention to perform a given behavior [14]. Ajzen [14] stated that the stronger the intention to engage in a behavior, the more likely should be its performance. Another classic model is the transtheoretical model, the usefulness of which has been investigated for decades [15]. The model assesses an individual's readiness to adopt a new healthier behavior and describes strategies or processes to guide the individual through the stages of change, from precontemplation to action and maintenance.

Abraham and Michie [16] developed a taxonomy of 26 generally applicable behavior change techniques. The behavior change techniques are based on 6 behavior change theories: information-motivation-behavioral skills model, theory of reasoned action, theory of planned behavior, social cognitive theory, control theory, and operant conditioning. This taxonomy is useful in determining which techniques are applied in apps that are aimed at changing lifestyle [13] and hence to identify techniques that have been used with success, to develop better apps in the future, and to identify potential gaps in literature.

The purpose of this review was to provide insight into how mobile apps can contribute to the promotion of healthy lifestyles among adolescents and students. We provide an overview of apps that have been developed (also) for adolescents or students with the aim of improving health, by promoting healthy nutrition, physical activity, and preventing overweight and obesity. For the apps that have been identified, we describe which theoretical mechanisms were applied during the development of the apps, and we summarize the lessons learned.

## Methods

### Inclusion and Exclusion Criteria

Only publications written in English were included. Furthermore, studies were included if (1) the primary component of the intervention involves a mobile app and this mobile app is already developed; (2) the intervention targets healthy nutrition, or physical activity, or overweight prevention; and (3) the focus is on adolescents and students (aged 12-25 years). Studies that also included people outside this age range (eg, 18-30 years) were included because these studies could provide valuable information regarding the primary target group. The articles were read carefully for age-specific information. The inclusion criteria for healthy nutrition include, among others, eating more vegetables and fruits, reducing soft drink or increasing water consumption, and reducing snack consumption. The applied methodology of the studies, for example, to assess effectiveness, was not an inclusion criterion because this was not relevant for answering parts 1 and 2 of the research questions (ie, the overview of existing apps and their theoretical bases). For the third part, the lessons learned, this is of relevance, and we mention the research methodology in that section of the paper.

Studies were excluded if researchers did not develop the mobile app described, if researchers did not focus on mobile apps, or if the app was used for data collection for research purposes (monitoring). Studies were included when data collection was applied with the intention of changing behavior. For example, the app served as a tool that was meant to increase awareness, a relevant step during the process of changing behavior. Other exclusion criteria were apps developed for a specific group of people with a health-related condition (patients with congenital heart disease or diabetes) and studies that focused on other preventive health issues, such as sport injuries and alcohol abuse.

### Search Strategy

**Figure 1:**
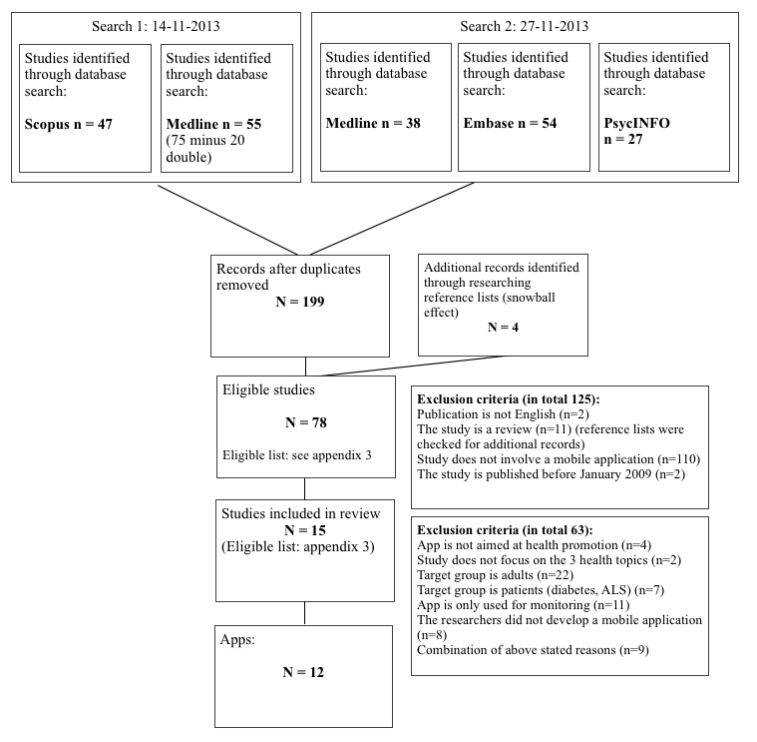
Search strategy.

The research databases Scopus, MEDLINE, Embase, and PsycINFO were searched for publications from January 2009 to November 2013 (Scopus and MEDLINE November 14, 2013; Embase, MEDLINE, and PsycINFO November 27, 2013). It was decided to include studies since January 2009 because the Apple App Store was opened in July 2008. For Scopus, the following search string was used: (((“mobile phone*” OR “smart phone*”OR “smart-phone*”) AND (“app” OR “apps” OR “application”)) AND (TITLE (physical* OR healthy OR overweight OR nutrition* OR exercise*))) AND (TITLE-ABS-KEY-AUTH (adolesc* OR young* OR school* OR teenager*)) AND (LIMIT-TO (DOCTYPE, “ar”) OR LIMIT-TO (DOCTYPE, “re”)). The following search terms were used for Embase, MEDLINE, and PsycINFO (November 27, 2013): smartphone (smart phone*, mobile phone*, game, games, gaming, mobile (all in title), phone, phones, android), exercise, sports, physical activity, food, body weight, nutrition, adolescent, young adult, youngster, teenage*, and mHealth. The extensive search strategy is shown in Multimedia Appendix 1. The first search in MEDLINE was slightly different from the second because it did not include the term game (games, gaming), fewer terms were used concerning health behaviors (sports, physical activity, nutrition), and it added the term telemedicine (Multimedia Appendix 2). These searches resulted in 199 unique studies (Figure 1). To obtain additional eligible studies, the reference lists of relevant studies and reviews were searched. As a result, 4 more studies were added. Figure 1 illustrates the search and selection process.

### Data Extraction and Synthesis

On the basis of title, 203 studies were screened. A total of 125 studies were excluded because they did not match the following criteria: the study was not written in English (n=2), the study was a review (n=11; reference lists were checked for additional studies), the article did not involve a mobile app (n=110), and the study was published before January 2009 (n=2). Thereafter, abstracts and, in some cases, entire studies were read. Studies were excluded based on target group (adults n=22, patients n=7), aim of the app (not part of a health promotion strategy n=4, only monitoring for research purpose n=11), topics addressed in the app (n=2), and a combination of these exclusion criteria (n=9). Furthermore, studies were excluded if the primary component of the intervention did not involve a mobile app or the mobile app was not developed by the researchers (n=8). Eventually 15 studies were included. Information of the included studies was extracted into a structured summary table. This table was not ordered by study, but by the apps, because this is the focus of this review. Some studies described several apps and some apps were described in several studies. Information of interest was information about the target group, study design, health topic, aim of the app, working mechanism of the app, mode of delivery, and use of theories and behavior change techniques (Tables 1 -3).

The taxonomy developed by Abraham and Michie [16] was applied to determine the use of behavior change techniques in the apps. Data were extracted from the app description in the studies by two researchers (DD and WB). Subsequently, these findings were discussed and are described in Table 3. For example, we decided to assign behavior change technique 12, “prompt self-monitoring of behavior,” when the app asked users to self-monitor their physical activity or daily intake. In all cases, this behavior technique appeared to be combined with behavior change technique 13, “provide feedback on performance,” because—according to Abraham and Michie—this is already the case when the app provides summaries about reported behavior. We considered it to be relevant to distinguish between providing summaries only and providing evaluative feedback concerning the reported behavior and whether this is in line with goals or guidelines or not; for example, the recommended amounts of fruit and vegetable intake per day. This distinction is indicated in Table 3. Also with respect to technique 10, “prompt specific goal setting,” we made a distinction and specified whether this concerned individual goal setting or whether goal setting was predefined and based on relevant public health guidelines. If an online buddy was part of the design of the app, then we allocated behavior change technique 20, “plan social support or social change.”

## Results

### Mobile App Characteristics

The 15 included studies describe 12 apps in total (Table 1). All studies were published since 2010 and Western research institutes developed the apps; 7 studies had been conducted in the United States, 4 in Australia, 2 in the United Kingdom, and 2 in Germany.

Two apps strove for increased physical activity levels by making the users aware of their daily amount of physical activity and by encouraging users to exercise. Eight apps aimed for dietary improvements, and 2 apps targeted both topics. Regarding dietary improvements, the apps focused on increased fruit and vegetable intake, reduced consumption of sugar-sweetened drinks, reduced excessive intake of fast food, monitoring all foods and beverages consumed, and defining problem foods.

Table 1 presents the reach and duration of usage of apps. Reach represents the number of users of apps. Some apps focused especially on participants at high risk of becoming overweight or obese (ePASS, eVIP, eSIYP, eTIYP) or participants who are overweight (W8Loss2Go, MoSeBo/DiaTrace). Four apps focused specifically on users aged 11-18 years (Ak-Shen, FRapp, App-Hongu, MoSeBo/DiaTrace) and some studies also included participants older than 25 years (MMM, ePASS, eVIP, eSIYP, eTIYP). The duration of app usage varied from 1 lunch and meal (CHAT) to 6 months (MMM).

Ten apps function as monitoring tools for assessing dietary intake or physical activity levels (Table 2). Assessing dietary intake is either done by an app that analyzes pictures taken before and after food and beverages are consumed by users (CHAT, MoSeBo/DiaTrace, Recaller, FRapp) or by self-monitoring what users have eaten (eVIP, eSIYP, eTIYP, MMM). In terms of physical activity, users can specify the type and intensity of the activity (ePASS, App-Hongu) or the apps measure the physical activity levels with a built-in sensor, such as a pedometer or accelerometer (MoSeBo/DiaTrace).

Two apps have a different approach. The Ak-Shen app uses a Web-based platform that challenges users to execute activities and to share this information with others. Users can upload this information with Global Positioning System (GPS) and camera features that are included in the app. W8Loss2Go focuses on compulsive overeating by allowing users to list and photograph their problem foods and to set targets in order to tackle this problem.

Regarding the broader context surrounding the apps, most apps are applied as part of a prevention program. The apps ePASS, eVIP, eSIYP, and eTIYP are part of TXT2BFiT, a healthy lifestyle program, which consists of a booklet, a Web site, community blogs, text messages, emails, and the apps to monitor several health conditions. The CHAT app is also part of a broader program in which students are supported by text messages. MoSeBo/DiaTrace is integrated in a structured treatment and teaching program (STTP). The app is used to assess physical activity levels and eating habits. W8Loss2Go can be used as part of a therapeutic program for obese children. Ak-Shen is implemented at school as part of a physical education class. App-Hongu contributes to a broader intervention strategy that promotes walking via a Web site containing a social element; that is, youth register as teams that can compete with each other. Other apps function on their own (MMM) or the broader context is not clearly described (Recaller, Frapp).

**Table 1:** Characteristics of mobile apps.

No.	Mobile app	Topic (nutrition, physical activity): aim of the app	Described in the following study/studies (first author, year, country)	Reach (participants' characteristics)	Duration of the usage
1	ePASS	Physical activity: increase physical activity level.	Hebden, 2012, Australia [ 17]	21 participants at high risk of becoming overweight or obese, 18-35 years old (10 of them evaluated the app).	Not described
			Hebden, 2013, Australia [ 18]	RCT^a^has not yet been performed, unknown.	RCT has not yet been performed, unknown
2	eVIP	Nutrition: increase fruit and vegetable intake.	Hebden, 2012, Australia [ 17]	21 participants at high risk of becoming overweight or obese, 18-35 years old (10 of them evaluated the app).	Not described
			Hebden, 2013, Australia [ 18]	RCT has not yet been performed, unknown.	RCT has not yet been performed, unknown
3	eSIYP	Nutrition: reduce consumption of sugar-sweetened drinks.	Hebden, 2012, Australia [ 17]	21 participants at high risk of becoming overweight or obese, 18-35 years old (10 of them evaluated the app).	Not described
			Hebden, 2013, Australia [ 18]	RCT has not yet been performed, unknown.	RCT has not yet been performed, unknown
4	eTIYP	Nutrition: reduce excessive intake of high-fat takeout (fast-food) meals.	Hebden, 2012, Australia [ 17]	21 participants at high risk of becoming overweight or obese, 18-35 years old (10 of them evaluated the app).	Not described
			Hebden, 2013, Australia [ 18]	RCT has not yet been performed, unknown.	RCT has not yet been performed, unknown
5	CHAT (Technology Assisted Dietary Assessment (TADA) Project)	Nutrition: increase fruit and vegetable intake, reduce junk food intake.	Kerr, 2012, Australia [ 19]	RCT has not yet been performed, unknown. Intention is to include users aged 18-30 years living in the suburbs of Perth, Western Australia.	RCT has not yet been performed, unknown
			Zhu, 2010, USA [ 20]	78 participants (26 males, 52 females), 11-18 years old.	Not described
			Six, 2010, USA [ 21]	Sample 1: 78 participants (26 males, 52 females), 11-18 years old. Sample 2: 15 participants, 11-18 years old.	Sample 1: one lunch and meal Sample 2: 1 day
			Six, 2011, Australia [ 22]	15 participants (12 boys, 3 girls), adolescents.	1 day
6	MoSeBo/DiaTrace	Nutrition, physical activity: weight reduction or stabilization.	Schiel, 2012, Germany [ 23]	124 participants (44% males, 56% females), average age 13.5 years.	On average 36.5 days
			Schiel, 2010, Germany [ 24]	30 overweight/obese participants, average age 14 years.	On average 4 days
7	Ak-Shen app (part of i-Challenge! program)	Nutrition, physical activity: increase physical activity, fruit and vegetable consumption, nutrition knowledge, motivation.	Mosqueda, 2012, USA [ 25]	30 healthy participants (21 males, 9 females), 11-14 years old.	8 weeks
8	MMM (My Meal Mate)	Nutrition: weight loss by self-monitoring of food and drink intake.	Carter, 2012, UK [ 26]	50 participants (students and staff).	7 days
			Carter, 2013, UK [ 27]	43 participants, 18-65 years old (66% males, 33% females).	6 months
9	Recaller^b^	Nutrition: raising awareness of dietary intake and eating pattern.	Suzuki, 2012, USA [ 28]	41 participants, college students (median age 22 years).	6 days
10	W8Loss2Go^c^	Nutrition: weight loss by identifying problem foods.	Pretlow, 2012, USA [ 29]	12 obese participants, 8-21 years old.	2 months
11	FRapp^c^	Nutrition: monitor dietary intake.	Casperson, 2013, USA [ 30]	17 participants, 11-14 years old.	3-7 days
12	App-Hongu^b^	Physical activity: encouraging reporting of miles walked in a physical activity program.	Hongu, 2013, USA [ 31]	30 participants, 11-14 years old.	Not described

^a^RCT: randomized controlled trial.

^b^Only a conference abstract was found and the authors did not respond to attempts to contact them.

^c^Only a conference abstract was found; studies are not yet published.

**Table 2:** Detailed information about mobile apps.

No.	Mobile app	Context	Short description of mobile app
1	ePASS	ePASS is part of the TXT2BFiT program, which consists of a booklet, a Web site, weight tracker, handouts, community blog, text messages, emails, personal coaching calls.	ePASS uses the target of moderate-level exercise for 30 minutes per day. Users can specify the type of activity and intensity and self-monitor their daily level of physical activity.
2	eVIP	eVIP is part of the TXT2BFiT program, which consists of a booklet, a Web site, weight tracker, handouts, community blog, text messages, emails, personal coaching calls.	eVIP allows users to monitor their daily intake of fruits and vegetables. A graphical display shows the number of fruits and vegetables the user recorded. As a reference, the app uses the targets of 2 servings of fruits and 5 servings of vegetables daily.
3	eSIYP	eSIYP is part of TXT2BFiT program, which consists of a booklet, a Web site, weight tracker, handouts, community blog, text messages, emails, personal coaching calls.	eSIYP allows users to specify the drink category (eg, water, tea or coffee, alcohol). The app presents users with a colored display with the total amounts of energy, sugar, and alcohol intake. The colors green, orange, and red indicate “ideal”, “acceptable”, and “too much” as threshold levels of intake, respectively.
4	eTIYP	eTIYP is part of the TXT2BFiT program, which consists of a booklet, a Web site, weight tracker, handouts, community blog, text messages, emails, personal coaching calls.	eTIYP allows users to specify the food and beverages consumed. A colored display shows the average energy and fat content of takeout meals, in which green indicates acceptable intake and red indicates excessive intake.
5	CHAT	CHAT used text messages to send users tailored feedback. Users are trained in using the app in advance and the app is currently developed to be used on an iPod touch.	CHAT provides users the ability to assess dietary intake (fruits, vegetables, junk food) by taking before and after pictures. Based on nutrition characteristics and volume estimation, tailored feedback and dietary recommendations are given regarding the estimated energy and nutrition.
6	MoSeBo/DiaTrace	The app is integrated in a structured treatment and teaching program (STTP) for overweight children and adolescents. The STTP consists of 28 therapeutic sessions in which personal goals are defined for each patient with respect to energy intake and physical activity.	The app consists of a built-in sensor that measures physical activity (mobile motion sensor, MoSeBo). The sensor measures the type, intensity, and duration of physical activity. The amount of physical activity is displayed on the display of the phone. With the camera (DiaTrace) eating habits are documented.
7	Ak-Shen app	i-Challenge! is an 8-week intervention that consists of an app and Web site and is part of a physical education class at junior high school. In a newsletter, a weekly i-Challenge! is delivered. An i-Challenge! is a small, fun, and challenging activity related to nutrition and physical activity intended to keep participants engaged in the project.	Ak-Shen app allows users to share activities with others. It consists of 3 components: 2 GPS-based mobile phone apps (GeoKnect and GeoSnap) and a social network, i-Challenge!. With GeoKnect and GeoSnap, the user can directly show on i-Challenge! what activity they do. With GeoKnect, a GPS-based feature, users can mark and describe points, lines, and areas of interest on a map. GeoSnap is a camera that captures photos and their descriptions and sends them automatically to the i-Challenge! Website.
98	MMM (My Meal Mate)	Users are also supported by tailored weekly text messages.	The MMM app allows users to set a weight loss goal and self-monitor daily calorie intake. Users select food and drinks consumed from a database and record items in an electronic food diary. Users can take photographs of their meals that serve as a memory aid. Physical activity can also be recorded in the diary.
9	Recaller	Not described.	Recaller is a nutrition assessment tool that allows users to take photos of all food eaten to improve diet awareness.
10	W8Loss2Go	Not described.	The app allows users to list and photograph their problem foods, with sequential withdrawal from each food. Furthermore, it includes a buddy and online community support.
11	FRapp	Not described.	FRapp is a food record app. It allows users to monitor dietary intake by taking before and after pictures of all foods and beverages consumed.
12	App-Hongu	The app is added to an 8-week online walking program that uses a Web site.	The app allows youth to report their walking miles.

### Theoretical Basis

Of the 12 apps, 5 described behavioral theories that served as a foundation for the apps (Table 3). The 4 apps of the TXT2BFiT program (apps 1-4) are based on the transtheoretical model, also called stages of change [15,17]. Hebden et al [17] describe the modeling process outlined in this model called self-reevaluation and may be of particular importance to progress from the contemplation to the action phase. Their apps use images of good examples, such as young adults looking healthy who are drinking water or riding a bike. Presenting role models who perform the target behaviors can motivate users to change their behavior. Another example of using the model by Hebden et al [17] is that users receive motivational tips as a source of positive encouragement to enhance self-efficacy.

CHAT (app 5) is based on the self-determination theory (SDT), combined with motivational interviewing [19]. The SDT states the importance of intrinsic motivation in order to change a person's behavior [19]. Critical aspects are autonomy, feeling competent to perform the behavior, and whether the person feels appreciated and understood by others [32]. In CHAT the SDT is combined with motivational interviewing because the tone and content of messages is supposed to be more effective in supporting autonomous decision making, as opposed to giving “traditional advices.” The app provides users the possibility to refuse information when not appreciated and sends tailored messages only. For example, a woman who does not drink alcohol does not receive any messages on alcohol consumption.

A behavior change theory, it is not specified which one, is applied in the development of the Ak-Shen app (app 7) [25]. This app is reported to focus on raising awareness and increasing motivation and provides tailored feedback to the users. Finally, the W8Loss2Go app (app 10) is not precisely based on a classic theory of changing behavior, but still the authors clearly underpin the theoretical base. They describe that the app focuses on problem foods and that coping skills are enhanced by showing other ways to handle negative emotions and neutralize cravings [29].

Subsequently, it was determined which behavior change techniques, identified by Abraham and Michie [16], were applied in the apps (Table 3). In a few studies, these techniques were clearly described, whereas in others they have been distilled out of the app descriptions given in the studies, as indicated in Table 3. The techniques “prompt self-monitoring of behavior” (technique 12) and “provision of feedback on performance” (technique 13) are most often applied, in 10 apps.

Another frequently applied technique is “specific goal setting” (technique 10), which is applied in 6 apps, mostly with general guidelines as being the reference for the target behavior (apps 1-4). As underpinned by several authors, it is important to provide contingent rewards (technique 14). These apps provide motivational tips (apps 1-4) or tailored messages (apps 5 and 8) based on the targeted behavior. The aim of these messages is to increase users' self-efficacy and to reinforce positive behavioral beliefs. Because the authors stress the importance of providing tailored feedback, possibly this technique is also applied in the Ak-Shen app (app 7).

Two apps primarily used other techniques than self-monitoring. The Ak-Shen app (app 7) provides adolescents with specific physical activity challenges (ie, treasure hunt, mapping, earth drawing, and tag) supported by the app, which incorporates a Global Positioning System. Furthermore, this app applies a technique involving social comparison. Users can share their activities with other members.

W8Loss2Go (app 10) allows users to list and photograph their problem foods in order to prompt barrier identification. The app aims at relapse prevention by providing coping skills, showing other ways to handle negative emotions and neutralize cravings. In line with Ak-Shen (app 7), this app uses a technique involving social support. Users are, for example, linked to a buddy. Furthermore, the MoSeBo app (app 6) virtually connects users with a buddy and provides the buddy information on performance, enhancing social comparison.

**Table 3:** Applied behavior change techniques in mobile apps.

No.	Mobile app	Theoretical basis	Behavior change techniques by Abraham and Michie [ 16] (In brackets is the number of the technique as numbered in the taxonomy, 2008).	Examples of applied behavior change techniques
1	ePASS	Transtheoretical model	Model or demonstrate behavior (9) Prompt specific goal setting (10) Prompt self-monitoring of behavior (12) Provide feedback on performance (13) Provide contingent rewards (14)	9: ePASS uses healthy role models. 10: ePASS shows the participants the recommended amount of daily physical activity (based on guidelines). 12: ePASS allows users to monitor their daily physical activity levels. 13: ePASS shows users the recorded amount of physical activity. 14: ePASS provides motivational tips.
2	eVIP	Transtheoretical model	Model or demonstrate behavior (9) Prompt specific goal setting (10) Prompt self-monitoring of behavior (12) Provide feedback on performance (13) Provide contingent rewards (14)	9: eVIP uses healthy role models. 10: eVIP shows the participants the recommended fruit and vegetable intake (based on guidelines). 12: eVIP allows users to monitor their fruit and vegetable intake. 13: eVIP provides (evaluative) feedback on the reported behavior. 14: eVIP provides motivational tips.
3	eSIYP	Transtheoretical model	Model or demonstrate behavior (9) Prompt specific goal setting (10) Prompt self-monitoring of behavior (12) Provide feedback on performance (13) Provide contingent rewards (14)	9: eSIYP uses healthy role models. 10: eSIYP shows the participants the allowed amount of sugar-sweetened drink intake. 12: eSIYP allows users to monitor their energy, sugar, and alcohol intake. 13: eSIYP shows users the recorded amount of energy, sugar, and alcohol intake (and values these amounts). 14: eSIYP provides motivational tips.
4	eTIYP	Transtheoretical model	Model or demonstrate behavior (9) Prompt specific goal setting (10) Prompt self-monitoring of behavior (12) Provide feedback on performance (13) Provide contingent rewards (14)	9: eTIYP uses healthy role models. 10: eTIYP shows the participants the allowed amount of take-out meals intake (based on guidelines). 12: eTIYP allows users to monitor their take-out meals intake. 13: eTIYP provides (evaluative) feedback on the reported behavior. 14: eTIYP provides motivational tips.
5	CHAT	Self-determination theory Motivational interviewing	Prompt specific goal setting (10) Prompt self-monitoring of behavior (12) Provide feedback on performance (13) Provide contingent rewards (14) Motivational interviewing (25)	10: CHAT sets goals based on dietary assessment. 12: CHAT allows self-assessment of dietary intake. 13: CHAT provides feedback on recorded nutrition performed (based on guidelines). 14: The participants receive messages to increase motivation. 25: Tone and content is carefully designed to enhance autonomous decision making and users can refuse to receive messages on particular content.
6	MoSeBo/DiaTrace	Not described	Prompt self-monitoring of behavior (12) Provide feedback on performance (13) Plan social support or social change (20)	12: MoSeBo/DiaTrace measures physical activity. 13: MoSeBo/DiaTrace displays the performed amount of physical activity. 20: Participants are virtually connected with a “buddy”. The current results of the buddy are shown on the app.
7	Ak-Shen app	Behavior change theory; not specified by the authors^a^	Set graded tasks (7) Provide instruction (8) Provide opportunities for social comparison (19)	7: The participants received four different challenges on their phones. 8: Information about the physical activities is delivered via the i-Challenge! social network. 19: The i-Challenge! social network is a virtual community that allows participants to upload their activities and share it with other members.
8	MMM (My Meal Mate)	Authors stress the importance of goal setting, self-monitoring, and feedback messages	Prompt specific goal setting (10) Prompt self-monitoring of behavior (12) Provide feedback on performance (13) Provide contingent rewards (14)	10: The app allows users to set weight loss goals. 12: Participants are asked to self-monitor their dietary intake. 13: The app displays daily calorie intake. 14: Feedback via tailored text messages weekly.
9	Recaller	Not described	Prompt self-monitoring of behavior (12) Provide feedback on performance (13)	12: The app provides the ability to monitor dietary intake. 13: The app provides feedback on dietary intake, based on photos that users take.
10	W8Loss2Go	Identification of problem foods and enhancing coping skills	Prompt barrier identification (5) Plan social support or social change (20) Relapse prevention (23)	5: The user is able to list and photograph his/her problem foods. 20: The app includes a buddy and online community support. 23: Relapse prevention is provided by determining problem food, increasing self-esteem, and coping skills augmentation.
11	FRapp	Not described	Prompt self-monitoring of behavior (12) Provide feedback on performance (13)	12: The app provides the ability to monitor dietary intake. 13: The app provides feedback on dietary intake.
12	App-Hongu	Not described	Prompt self-monitoring of behavior (12) Provide feedback on performance (13)	12: The participants registered the miles walked with their mobile phones. 13: The app directly shows the results on a Web site.

^a^The authors indicate that the app raises awareness, increases motivation, and provides tailored feedback.

### Lessons Learned

To make statements regarding the lessons learned, it is important to know which research methods were used to test the apps. Only 2 studies measured the effect of using the app on the target group: Schiel et al [23] and Mosqueda [25]. Hebden et al, 2013 [18] and Kerr, 2012 [19] described a protocol for a randomized controlled trial (RCT), for assessing the impact in the future. At this moment, lessons learned are known only from pretesting the app among the target population, as is the case for the other studies (see Table 1 , column “Reach,” for a description of the target population).

Schiel et al [23] (MoSeBo) reported a significant weight reduction among users of the app but made no comparison to a control group. Furthermore, these participants participated in an STTP and the precise contribution of the app has not been specifically investigated. The researchers stated that the app was successful at improving the intrinsic and extrinsic motivation of the participants.

Mosqueda [25] (Ak-Shen) performed a nonrandomized experiment suggesting the potential of a smartphone-based intervention. No significant changes were found between the app group and control group, but baseline characteristics differed, such as age, height, weight, and ethnicity, and the sample size was small (30 adolescents).

In several studies, the pretests indicated that the users were willing and able to use the app. The users of CHAT (app 5) aged 11-18 years considered the software easy to use, and no difference in proficiency with the tool was found between users aged 11-14 years and 14-18 years [21]. The Recaller (app 9) was extremely easy or easy to use, according to participants [28]. Hongu et al [31] (app 12) stated that the “mobile phone group” had a higher response rate compared with the “website group,” suggesting a useful contribution of the app. Pretlow and Gearhardt [29] (W8Loss2Go) stated that the app was understandable to all participants, except for those under age 10 years. This would mean that we found no evidence that apps may be feasible for users under the age of 10 years, because their study included children aged 8-9 years.

Five of the included apps (apps 1-4 and app 8; described in 2 studies) also specified users outside the age range as their target population, that is, people older than 25 years. According to apps 1-4, qualitative feedback provided by young adults related to practical features such as the speed of using the app and the necessity of a login, which was considered a barrier.

Duration of usage varies largely among the apps, which makes it difficult to make statements regarding feasibility of long-term use. Some apps were only tested for several days: CHAT (app 5) was used for 1 day, Recaller (app 9) for 6 days, and FRapp (app 11) for 3-7 days [21,28,30]. Other apps were used for a longer period: the MoSeBo/DiaTrace (app 6) on average for 36.5 days, the Ak-Shen app (app 7) for 8 weeks, MMM (app 8) for 6 months, and W8Loss2Go (app 10) for 2 months [23,25,28,29].

## Discussion

### Key Findings

Limited research has been done so far on mobile apps and their use in health promotion for adolescents and students. This review found only 15 studies that describe the use of 12 apps to improve the health of adolescents and students regarding their dietary intake and physical activity levels. This may seem surprising because apps are widely used, especially among adolescents and students; 23% of the European adolescents download free apps on a daily basis [33]. Furthermore, in recent years many apps have been developed. Breton et al [34] found 204 apps for weight control on iTunes on September 25, 2009, and Conroy et al [4] found 167 top-ranked apps for physical activity in 2013. However, according to an appropriate description of apps in scientific literature, the number is very disappointing, and obviously, this limits the possibilities for an in-depth discussion of the advantages and disadvantages of using certain theories or techniques of changing behavior in apps.

The limited number of publications concerning apps indicates the difficulty of capturing technology in science. Because of the dynamic and rapid development of apps and the long processes of doing research and publishing, it is difficult to provide up-to-date information. The MoSeBo/DiaTrace app by Schiel et al [23] serves as an example. Although they published about 2 years ago, the mobile phone they used seems to be outdated (based on author's evaluation of the absence of a touchscreen). It should be recognized that it is therefore more difficult to make statements corresponding to the current situation of app development.

For 5 of the 12 included apps, the behavioral theory underpinning their development was clearly specified. The 4 apps developed by Hebden et al [17,18] used the transtheoretical model, which is also called stages of change [15,17]. The apps use role models who perform the target behavior, which can motivate users to change their behavior. CHAT is based on the SDT and supports autonomous decision making by the users. It is of interest that one of the included apps in our review that did not primarily focus on self-monitoring, that is, the W8Loss2Go app, has a theoretical base in addiction-derived theories. W8Loss2Go targets compulsive overeating, which is considered as addictive behavior, by barrier identification, offering social support, and relapse prevention. The authors did not specify the used theory in detail. More research should be carried out in this area, because the available evidence is sparse.

We applied the taxonomy of behavior change techniques [16] to the identified apps for this review. It became clear that healthy diets and physical activity are mainly promoted by means of self-assessment tools. Out of 12 apps, 10 were designed either to monitor nutrition intake or to monitor duration or the intensity of physical activities. These apps applied behavior change techniques such as prompting self-monitoring and providing feedback on performance. By facilitating self-monitoring and providing feedback on performance, apps can increase awareness about the dietary intake and physical activity levels of the users. This suggests that apps can be suitable as a monitoring tool, which is underpinned by Breton et al [34] who found that of 204 apps included in their review, 43% provided a food diary tool.

Besides the behavior change techniques of self-monitoring and providing feedback on performance, specific goal setting combined with personal feedback messages is also considered as a promising approach. Kohl et al [11] also concluded that these approaches, goal setting and personal feedback, are effective elements in Web-delivered interventions. High-quality evidence supporting effectiveness of these apps, however, is not yet available. A pilot randomized trial of Carter et al [27] found significant weight reduction among the app users. Although the app is included in this review because the target population involves students, the RCT participants consisted of adults, most of them older than 25 years. This result may also apply to students, and because we found no strong arguments counteracting this, for example, from the process evaluation, we consider this a promising result. Besides the significant weight reduction, trial retention and adherence were also significantly higher in the smartphone group compared with the other groups. These results indicate the potential of applying these behavior change techniques in apps to improve the health of adolescents and students; however, no conclusions can be drawn regarding the effectiveness of these apps.

Three apps (MoSeBo/DiaTrace, Ak-Shen, and W8Loss2Go) offer a social function and can be categorized as social media. An example is the Ak-Shen app that uses an online platform that challenges users to execute activities and to share this information with others. The app contributes to users' motivation by providing rewards and opportunities for social comparison. Kaplan and Haenlein [35] describe that research into social media should acknowledge features like social presence and media richness, and social processes, which includes self-presentation and self-disclosure. As there is a clear gap in literature, it would be valuable to conduct more research into social features in mobile apps and their effectiveness in health promotion for adolescents and students. Several apps provided social comparison opportunities or a buddy system, but the behavior change technique “provide information on others' approval” did not appear to be specifically applied.

It should be clarified that we described techniques (summarized in Table 3) that were applied in the apps only, although almost all the included apps were or can be implemented as part of a broader intervention strategy. It probably explains why behavior change techniques, like providing information on the link between behavior and health or providing general encouragement, were not clearly present in the app descriptions. These techniques are probably applied within the broader intervention strategy, strengthening the potential effect of the app. This is in line with Crutzen et al [10] who stated that apps are more effective when they are embedded in existing structures, such as health care and schools.

According to the technique “teach about environmental cues” (15), we identified one app demonstrating this potential [36]. However, this app was not included in this review because it is used primarily for research purposes (monitoring), instead of changing behavior. Regarding apps for adolescent, gaps in literature seem to be in using techniques such as “(follow-up) prompts of practice” (18) or “review of goals” (11). More research is recommended on the application of behavior change techniques among adolescents and students.

Mobile apps have the potential to tackle health issues. Several arguments are mentioned in the studies. Hebden et al [17] stated that the features of apps—widespread and increasing use, dynamic technologies, and use of existing features like Internet, cameras, and GPS—make them potentially able to improve health. Apps are easy to use and to access [17,34]. Carter et al [27] have underpinned the advantages as compared with traditional methods (“paper diaries”). Obviously, by linking apps to databases, and subsequently providing feedback on energy intake (calories) or energy expenditure based on self-monitoring, useful feedback is possible in an efficient way, which enhances awareness and goes beyond the registration of food intake only.

Our review not only supports the idea that apps can be a promising tool as part of health promotion strategies but also highlights that the scientific base for adolescents is small. Hardly any evidence on their effectiveness is available from high-quality research studies. Even less evidence is available for longer-term usage, which is supposed to enhance maintenance of any changes in behavior that may be achieved when using the app. Most apps are tested on a small scale only and for a short period. It is unclear for what duration the users are willing to test the apps. The MMM app (app 8) was used for 6 months (which is the longest period), but within the context of an RCT mainly involving adults [27]. The average number of adherence days was 92 in the smartphone group, as compared with 35 days and 29 days in the other two groups [27]. Furthermore, only 5 out of 12 apps described a theoretical basis for the app, and several promising techniques had either not yet been applied or only been applied in a few apps. Finally, it is of particular interest to study whether a social aspect contributes to the effectiveness of mobile apps. In general, the importance of social media for young people, for example, regarding self-presentation, self-disclosure in social processes, shaping their identity, and so on, seems to be worth exploring more for health promotion strategies.

### Strengths and Limitations

The strength of this review is that it approaches a new area of research in which there is still much to discover and to learn. Although the search revealed a large number of hits, as summarized in appendix 3 , only a limited number of studies met the inclusion criteria. Only two studies investigated the effect of using the apps, so it was impossible to perform a systematic review and meta-analysis on effectiveness. However, the studies did provide interesting information that could be used when designing a new mobile app. Five studies had not yet been published in a peer-reviewed journal, and information about the app was derived from an abstract [28-31] and provided by the authors [25]. Hence, this review provides an overview of the current scientific “state of the art” in this rapidly developing field and presents some upcoming studies.

Overall, it is useful to have used the taxonomy of techniques for this review, because it clearly demonstrates the focus of these apps. It should be noted, however, that this taxonomy is primarily proposed for health promotion in adults. Some techniques, which may be particularly important for young people, could not clearly be coded; for example, the incorporation of a game or competition element. This was not part of the Hongu-app itself [31], but it was part of the broader intervention strategy, since the winning teams received a reward. With respect to the technique “demonstrate behavior by expert” (technique 9), not necessarily experts but also peers may be of relevance to youth; we coded several apps, using peers, by applying this technique (see Table 3).

Furthermore, inaccuracies may have occurred by applying the taxonomy of behavior change techniques [16] because of several reasons. Some of the apps were poorly described, and in a few situations, the same mobile app was described in more than one study. In that case, adjustments were made over time, possibly, but these were not always clearly described. An explanation for the large variation in description of the apps is the difference in research focus. Several studies were protocols for RCTs, others described the development of the app, and in some, entire intervention programs were described. Together with the rapid development of mobile apps, as described in the beginning of this discussion, it is questionable whether a review or traditional research methodology in general is the most appropriate method to gather information concerning mobile apps. Research with a qualitative methodology, capturing context and changing developments not only regarding technological developments but also regarding rapidly changing trends and hypes among young people, may be suitable as well.

Many different terms are used to describe mobile apps: smartphone applications/smart phone applications, mobile phone-based healthy lifestyle program, using mobile devices/mobile phones, electronic health technology, and a mobile telephone record; this makes the search for relevant studies very difficult. It is because of this that the two searches in MEDLINE were not entirely consistent. As a result of a small adjustment in the research terms, not all studies found in search 1 were also included in search 2. Therefore, we decided to use both searches and to remove duplicates. It appeared necessary to use a broad definition for mobile apps. Some studies did not seem to describe a “real” mobile app. An example is the application of Schiel et al [23,24], which is implemented on an outdated mobile phone type that could be questioned as being a smartphone. However, after consideration, it was decided to include the app for this review because it met the other criteria and it provided valuable information, including being an illustration of the apparent mismatch between fast developments in technological possibilities as compared to scientific research, which is a slower process.

### Conclusions

The present review aimed to identify mobile apps to be used in health promotion for adolescents and students. It became clear that apps are suitable as monitoring tools for both dietary intake and physical activity. The apps enable users to set targets and self-monitor, provide tailored feedback, and subsequently raise awareness and increase motivation. These types of monitoring tools can function independently, but most of the identified apps are part of a treatment program or support educational methods, that is, methods used in schools. Three apps facilitated social interaction and support and can be characterized as social media. Subsequently, these “social” apps apply other behavior change techniques, such as providing opportunities for social comparison and social support, in comparison with monitoring apps. The limited number of studies clearly indicates the need for additional research, and one may question whether this should be performed in a “traditional” way. Further research is recommended on the effectiveness, reach, and long-term use of mobile apps and to identify other possibilities to tackle health issues with mobile apps, especially with respect to their potential social features.

## Appendix

Multimedia Appendix 1 Search strategy Embase, MEDLINE, and PsycINFO, November 27, 2013.

Multimedia Appendix 2 Search strategy MEDLINE, November 14, 2013.

Multimedia Appendix 3 Eligible table.

## Abbreviations

GPS: Global Positioning System
RCT: randomized controlled trial
SDT: self-determination theory
STTP: structured treatment and teaching program
