# Supplementary material for: Using Mobile Apps to Promote a Healthy Lifestyle Among Adolescents and Students: A Review of the Theoretical Basis and Lessons Learned
Source: JMIR Mhealth Uhealth. 2016 May 5;4(2):e39. doi: 10.2196/mhealth.3559 (PMC4873621; doi:10.2196/mhealth.3559)
Supplement: Multimedia Appendix 1 [file mhealth_v4i2e39_app1.pdf]

Appendix 1: Search strategy Embase, Medline & PsycINFO 27-11-2013

```
      NO      HITS  SEARCH EXPRESSION
-- ----- ----------
--------------------------------------------------------
C=     125810846  ME00; EM00; PI67
S=     2  4514    TI=SMARTPHONE? OR TI=SMART PHONE? OR TI=MOBILE PHONE? OR
TI=ANDROID?
       3  18489   TI=GAME OR TI=GAMES OR TI=GAMING
       4  16124   TI=MOBILE?
       6213758    CT DOWN EXERCISE
       7120395    CT DOWN SPORT
       8136526    CT DOWN SPORTS
       9302364    CT DOWN PHYSICAL ACTIVITY
      10868101    CT DOWN FOOD
      11470901    CT DOWN BODY WEIGHT
      12874443    CT DOWN NUTRITION
      132067944   6 OR 7 OR 8 OR 9 OR 10 OR 11 OR 12
      17  9328    AB=SMARTPHONE? OR AB=SMART PHONE? OR AB=MOBILE PHONE? OR
AB=ANDROID?
      19 94765    S=6/W=1
      20      `68454     S=7/W=1
      21 68454    S=8/W=1
      22112407    S=9/W=1
      23469027    S=10/W=1
      24118259    S=11/W=1
      25338184    S=12/W=1
      26929361    19 OR 20 OR 21 OR 22 OR 23 OR 24 OR 25
      291016737   AB=APP OR AB=APPS OR AB=APPLICATION? OR AB=GAME OR
AB=GAMES OR AB=GAMING
      3318785779  PPS=HUMAN
      362126030   ADOLESC? OR YOUNG ADULT? OR YOUNGSTER?
      37 30362    TEENAGE?
      382130644   36 OR 37
      45   444    MHEALTH?
      46  2001    29 AND (2 OR 17)
      47  2377    46 OR 45
      48   116    47 AND 26
      49  2550    3 AND 13
      50     8    49 AND 17
      51 33563    PHONE OR PHONES
      52    15    51 AND 49
      53   127    48 OR 50 OR 52
      54   124    33 AND 53
      55    98    check duplicates: unique in s=54

Additional search in PsycINFO 27-11-2013
C=     13591189   PI67
S=     2   114    TI=SMARTPHONE? OR TI=SMART PHONE? OR TI=ANDROID?
       3   500    TI=MOBILE PHONE?
       4   612    2 OR 3
       5 87888    TI=EXERCISE? OR TI=ACTIVIT? OR TI=OVERWEIGHT? OR
TI=SPORT? OR TI=PHYSICAL?
       6    17    4 AND 5
       7  8781    TI=GAME OR TI=GAMES OR TI=GAMING
       8   371    7 AND 5
       9150264    PHONE? OR APP OR APPS OR APPLICATION?
```

10	14	8 AND 9
11	31	6 OR 10
