# Supplementary material for: Using Mobile Apps to Promote a Healthy Lifestyle Among Adolescents and Students: A Review of the Theoretical Basis and Lessons Learned
Source: JMIR Mhealth Uhealth. 2016 May 5;4(2):e39. doi: 10.2196/mhealth.3559 (PMC4873621; doi:10.2196/mhealth.3559)
Supplement: Multimedia Appendix 2 [file mhealth_v4i2e39_app2.pdf]

Appendix 2: Search strategy Medline 14-11-2013

Database: MEDLINE 1950 to present
Search Strategy:
----
1 exp Cellular Phone/ (4574)
2 (app or apps or application$).ti,ab. (633523)
3 1 and 2 (675)
4 exp Exercise/ (115313)
5 exp Food/ (1068766)
6 exp Body Weight/ (354226)
7 (smartphone$ or (smart adj phone$) or (mobile adj phone$)).ti,ab. (3109)
8 exp Telemedicine/ (15602)
9 exp Internet/ (53914)
10 3 and 4 (15)
11 3 and 5 (9)
12 3 and 6 (23)
13 10 or 11 or 12 (41)
14 7 and 4 (61)
15 7 and 5 (51)
16 7 and 6 (83)
17 14 or 15 or 16 (178)
18 8 and 4 (78)
19 8 and 5 (19)
20 8 and 6 (130)
21 18 or 19 or 20 (201)
22 9 and (4 or 5 or 6) (1406)
23 exp *Internet/ and (exp *Exercise/ or exp *Food/ or exp *Body Weight/) (465)
24 2 and 23 (19)
25 exp adult children/ or exp adolescent/ or exp young adult/ (1788957)
26 13 and 25 (16)
27 17 and 25 (48)
28 21 and 25 (33)
29 24 and 25 (1)
30 22 and 25 (305)
31 30 and (2 or 7) (13)
32 31 not (26 or 27 or 28) (7)
33 26 or 27 or 28 (83)
34 mhealth$.ti,ab. (127)
35 34 and (4 or 5 or 6) (6)
36 35 and 25 (2)
37 33 or 36 (84)
38 limit 37 to yr="2009 -Current" (75)
