# Supplementary material for: Using Mobile Apps to Promote a Healthy Lifestyle Among Adolescents and Students: A Review of the Theoretical Basis and Lessons Learned
Source: JMIR Mhealth Uhealth. 2016 May 5;4(2):e39. doi: 10.2196/mhealth.3559 (PMC4873621; doi:10.2196/mhealth.3559)
Supplement: Multimedia Appendix 3 [file mhealth_v4i2e39_app3.pdf]

Appendix 3: Eligible table

Nr.	Title	First Author, year of publication, country	Database	Included	Reason for exclusion
1	Comparison of traditional versus mobile app self-monitoring of physical activity and dietary intake among overweight adults participating in an mHealth weight loss program	Turner-McGrievy, 2013, USA	Scopus & Medline	no	Target group are adults
2	SMART MOVE - a smartphone-based intervention to promote physical activity in primary care: Study protocol for a randomized controlled trial	Glynn, 2013, Ireland	Scopus	no	Target group are adults
3	TXT2BFiT' a mobile phone-based healthy lifestyle program for preventing unhealthy weight gain in young adults: Study protocol for a randomized controlled trial	Hebden, 2013, Australia	Scopus & Medline	yes	App is also described in Hebden et al (2012)
4	Mobile games and design requirements to increase teenagers' physical activity	Arteaga, 2012, USA/Mexico	Scopus	no	The researchers have not developed an app
5	A comparative study of pattern recognition classifiers to predict physical activities using smartphones and wearable body sensors	Kouris, 2012, Greece	Scopus	no	Only basic monitoring data
6	Children's perceptions of physical activity environments captured through ecological momentary assessment: A validation study	Dunton, 2012, USA	Scopus	no	Aim is not health promotion
7	Development of smartphone applications for nutrition and physical activity behavior change	Hebden, 2012, Australia	Scopus	yes	App is also described in Hebden et al (2013)
8	Connecting Health and Technology (CHAT): Protocol of a randomized controlled trial to improve nutrition behaviours using mobile devices and tailored text messaging in young adults	Kerr, 2012, Australia	Scopus	yes	App is also described in Zhu et al. (2010) and Six et al. (2010; 2011)

9	Recognition of physical activities in overweight Hispanic youth using KNOWME networks	Emken, 2012, USA	Scopus & Medline	no	Only basic monitoring data
10	Design and rationale for the PREVAIL study: Effect of e-Health individually tailored encouragements to physical exercise on aerobic fitness among adolescents with congenital heart disease - A randomized clinical trial	Klausen, 2012, Denmark	Scopus	no	Target group are "patients" (congenital heart disease)
11	Investigating the impact of a smart growth community on the contexts of children's physical activity using Ecological Momentary Assessment	Dunton, 2010, USA	Scopus & Medline	no	Aim is not health promotion
12	Desired features of smartphone applications promoting physical activity.	Rabin, 2011, USA	Scopus	no	Target group are adults
13	Investigating children's physical activity and sedentary behavior using ecological momentary assessment with mobile phones	Dunton, 2011, USA	Scopus & Medline	yes	Only basic monitoring data
14	Let's get technical! Gaming and technology for weight control and health promotion in children.	Baranowski, 2012, USA	Medline	no	The researchers have not developed an app
15	A smartphone-supported weight loss program: design of the ENGAGED randomized controlled trial.	Pellegrini, 2012, USA	Medline	no	Target group are adults
16	Is there a valid app for that? Validity of a free pedometer iPhone application.	Bergman, 2012, USA	Medline	no	Only basic monitoring data
17	Multiple behavior changes in diet and activity: a randomized controlled trial using mobile technology.	Spring, 2012, USA	Medline	no	Target group are adults
18	Comparison of known food weights with image-based portion-size automated estimation and adolescents' self-reported portion size.	Lee, 2012, USA	Medline	no	Target group are diabetic patients, Only basic monitoring data
19	Validity of the Remote Food Photography Method (RFPM) for estimating energy and nutrient intake in near real-time.	Martin, 2012, USA	Medline	no	Target group are adults Only basic monitoring data

20	Electronic health technology for the assessment of physical activity and eating habits in children and adolescents with overweight and obesity IDA.	Schiel, 2012, Germany	Medline	yes	App is also described in Schiel et al. (2010)
21	Assessing the social and physical contexts of children's leisure-time physical activity: an ecological momentary assessment study.	Dunton, 2012, USA	Medline	no	Aim is not health promotion
22	Consumer views on the potential use of mobile phones for the delivery of weight-loss interventions.	Gorton, 2011, New Zealand	Medline	no	Target group are adults The researchers have not developed an app
23	Feasibility testing of an automated image-capture method to aid dietary recall.	Arab, 2011, USA	Medline	no	Target group are adults Only basic monitoring data
24	Preliminary application of a new bolus insulin model for type 1 diabetes.	Pelzer, 2011, South Africa	Medline	no	Target group are diabetic patients
25	Intelligent obesity interventions using Smartphones.	Wolfenden, 2010, Australia	Medline	no	The researchers have not developed an app
26	Identification of determinants for weight reduction in overweight and obese children and adolescents.	Schiel, 2010, Germany	Medline	yes	App is also described in Schiel et al. (2012)
27	Evaluation of a mobile phone-based diet game for weight control.	Lee, 2010, South Korea	Medline	no	Target group are adults
28	Interactive diary for diabetes: A useful and easy-to-use new telemedicine system to support the decision-making process in type 1 diabetes.	Rossi, 2009, Italy	Medline	no	Target group are diabetic patients
29	I-Challenge!, A nutrition and physical activity intervention using smart phones in physical education classes at a junior high school	Mosquedo, 2012, USA	Embase	yes	
30	A pilot study of a novel mhealth intervention to improve complementary feeding practices in	Singh, 2013, UK	Embase	no	Target group is mothers and their infants

	Haryana State, India				
31	Adherence to a smartphone application for weight loss compared to website and paper diary: pilot randomized controlled trial.	Carter, 2013, UK	Medline	yes	App is also described in Carter et al. (2012)
32	BALANCE (Bioengineering Approaches for Lifestyle Activity and Nutrition Continuous Engagement): developing new technology for monitoring energy balance in real time.	Hughes, 2010, USA	Medline	no	Target group are adults
33	Design, development, and formative evaluation of a smartphone application for recording and monitoring physical activity levels: the 10,000 Steps "iStepLog".	Kirwan, 2013, Australia	Medline	no	Target group are adults Only basic monitoring data
34	Development of an automated physical activity classification application for mobile phones.	Xia, 2011, Australia	Medline	no	Target group are adults Only basic monitoring data
35	Digital food guide: Mobile application to incorporate healthy food choices	Caivano, 2013, Brazil	Embase	no	Target group are adults
36	Do arabic weight loss mobile applications comply with evidence-based weight management guidelines?	Alnasser, 2013, UK	Embase	no	Target group are adults
37	Food photo records for assessing daily food intake using a smart phone application in college students	Suzuki, 2012, USA	Embase	yes	Only abstract available
38	Implementing the dietary guidelines for Americans using new technologies as a channel for nutrition education: Evidence from research and best practices	Blum-Kemelor, 2012, USA	Embase	no	The researchers have not developed an app
39	Mobile applications for weight management: Theory-based content analysis	Azar, 2013, USA	Embase	no	The researchers have not developed an app
40	Mobile apps for pediatric obesity prevention and treatment, healthy eating, and physical activity promotion: Just fun and games?	Schoffman, 2013, USA	Embase	no	The researchers have not developed an app
41	My Meal Mate (MMM): Validation of the diet measures captured on a	Carter, 2012, UK	Embase	yes	App is also described in Carter et al.

	smartphone application to facilitate weight loss				(2013)
42	Self-assessment of the daily food intake in ALS via an application on a mobile device	Holm, 2011, Australi a	Embase	no	Target group are ALS patients
43	Smartphone application for real-time dietary assessment and physical activity analyses in dietary counseling	Beer-Borst, 2011, Switzerl and	Embase	no	Target group are adults
44	Substance dependence (problem food) approach to childhood obesity, implemented as a smartphone APP: A pilot study	Pretlow, 2012, USA	Embase	yes	Not yet published
45	Technology for health: A qualitative study on barriers to using the ipad for diet change	Lister, 2013, USA	Embase	no	The researchers have not developed an app
46	Usability of mobile phone food records to assess dietary intake in adolescents	Casperso n, 2013, USA	Embase	yes	Not yet published
47	Using smartphone technology to monitor physical activity in the 10,000 Steps program: a matched case-control trial.	Kirwan, 2012, Australi a	Medlin e	no	Target group are adults
48	Using smartphones to maintain engagement of youth participation in 8-week walking program	Hongu, 2013, USA	Embase	yes	Only abstract available
49	Validity and reliability of a smart phone-based physical activity monitoring application	Gravante , 2012, UK	Embase	no	Target group are adults
50	Weight loss-there is an app for that! But does it adhere to evidence-informed practices?	Breton, 2011, USA	Embase	no	The researchers have not developed an app
51	A randomized clinical trial of mhealth supported exercise intervention in patients with metabolic syndrome	Gill, 2013, Canada	Embase	no	Target group have metabolic syndrome
52	Activity recognition with android phone using mixture-of-experts co-trained with labeled and unlabeled data.	Lee, 2014, South Korea	PsycIN FO	no	Only basic monitoring data
53	Can mobile phones be used to routinely monitor nutrition indicators? Experience from Liberia	Guyon, 2013, USA	Embase	no	Only basic monitoring data

54	Classification accuracies of physical activities using smartphone motion sensors.	Whu, 2012, USA	PsycINFO	no	Only basic monitoring data
55	Comparison of physical activity measures using mobile phone-based CalFit and actigraph.	Donaire-Gonzalez, 2013, Spain	PsycINFO	no	Target group are adults Only basic monitoring data
56	Design and rationale for the PREVAIL study: Effect of e-Health individually tailored encouragements to physical exercise on aerobic fitness among adolescents with congenital heart disease - A randomized clinical trial	Klausen, 2012, Denmark	Embase	no	Target group are patients with congenital heart disease
57	Education, reregistration, and recommendation effect of iPhone Poomsae education app in Taekwondo academy.	Ha, 2011, South Korea	Embase	no	Aim is not health promotion
58	Harnessing Different Motivational Frames via Mobile Phones to Promote Daily Physical Activity and Reduce Sedentary Behavior in Aging Adults	King, 2013, USA	Embase	no	Target group are adults
59	Innovation to motivation-pilot study of a mobile phone intervention to increase physical activity among sedentary women.	Fukuoka, 2010, USA	PsycINFO	no	Target group are adults
60	Iterative development of MobileMums: a physical activity intervention for women with young children.	Fjeldsoe, 2012, Australia	Medline	no	Target group are adults
61	iWander: An Android application for dementia patients.	Sposaro, 2010, USA	Medline	no	Target group are adults
62	Measuring physical activity in a cardiac rehabilitation population using a smartphone-based questionnaire.	Pfaeffli, 2013, New Zealand	PsycINFO	no	Target group are patients with cardiac problems
63	Nigerian women participating in an integrated microcredit and mhealth breastfeeding promotion intervention were more likely to adopt international breastfeeding recommendations	Flax, 2013, USA	Embase	no	Target group are adults
64	Physical activity monitoring and sharing platform for manual wheelchair users.	Ding, 2012, USA	Medline	no	Only basic monitoring data
65	Promoting behavior change from alcohol use through	Cohn, 2011,	Embase	no	Topic is use

	mobile technology: The future of ecological momentary assessment	USA			
66	Qualitative exploration of the acceptability of a mobile phone and pedometer-based physical activity program in a diverse sample of sedentary women.	Fukuoka, 2012, USA	PsycINFO	no	Target group are adults
67	Reliability and validity of gait analysis by android-based smartphone.	Nishiquchi, 2012, Japan	Embase	no	Target group are adults Only basic monitoring data
68	The impact of the application "iNST" development for nutrition support for patients in Japan	Ino, 2012, Japan	Embase	no	Target group are patients and adults
69	The mPED randomized controlled clinical trial: applying mobile persuasive technologies to increase physical activity in sedentary women protocol.	Fukuoka, 2011, USA	Medline	no	Target group are adults
70	The TiltMeter app is a novel and accurate measurement tool for the weight bearing lunge test	Williams, 2013, Australia	Embase	no	Focus is not on nutrition, PA or overweight.
71	Towards the run and walk activity classification through step detection--an android application.	Oner, 2012, USA	Medline	no	Only basic monitoring data
72	Usability and feasibility of mobile phone diaries in an experimental physical exercise study.	Heinoner, 2012, Finland	Medline	no	Only basic monitoring data
73	Use of a mobile phone diary for observing weight management and related behaviours.	Mattila, 2010, Finland	Medline	no	Target group are adults
74	Using mHealth technology to enhance self-monitoring for weight loss: a randomized trial.	Burke, 2012, USA	Medline	no	Target group are adults
75	The use of mobile devices in aiding dietary assessment and evaluation	Zhu, 2010, USA	Snowball (Lee, 2012, Medline search 1)	yes	App is also described in Six et al. (2010; 2011) and Kerr et al. (2012)
76	Evidence -based development of a mobile telephone record	Six, 2010, USA	Snowball (Lee, 2012, Medline	yes	App is also described in Zhu et al. (2010), Six et al (2011) and Kerr et al.

			search 1)		(2012)
77	Evaluation of the food and nutrient database for dietary studies for use with a mobile telephone record	Six, 2011, USA	Snowball (Lee, 2012, Medline search 1)	yes	App is also described in Zhu et al. (2010) and Kerr et al. (2012), Six et al. (2010)
78	Personal dietary assessment using mobile devices	Mariappan, 2009, USA	Snowball (Lee, 2012, Medline search 1)	no	Only basic monitoring data
